# Development of Self-Healable Organic/Inorganic Hybrid Materials Containing a Biobased Copolymer via Diels–Alder Chemistry and Their Application in Electromagnetic Interference Shielding

**DOI:** 10.3390/polym11111755

**Published:** 2019-10-25

**Authors:** Yi-Huan Lee, Wen-Chi Ko, Yan-Nian Zhuang, Lu-Ying Wang, Tao-Wei Yu, Shaio-Yen Lee, Tun-Fun Way, Syang-Peng Rwei

**Affiliations:** 1 Institute of Organic and Polymeric Materials, National Taipei University of Technology, Taipei 10608, Taiwan; asd14120asd@gmail.com (W.-C.K.); nickhamesome@gmail.com (Y.-N.Z.); ying3650@gmail.com (L.-Y.W.); f10714@ntut.edu.tw(S.-P.R.); 2 Department of Molecular Science and Engineering, National Taipei University of Technology, Taipei 10608, Taiwan; 3 Research and Development Center for Smart Textile Technology, National Taipei University of Technology, Taipei 10608, Taiwan; tfway1951@gmail.com; 4 Taiwan Graphene Co. Ltd., Taipei 11493, Taiwan; bob.yu@angstronmaterials.com (T.-W.Y.); shaoyen.lee@angstronmaterials.com (S.-Y.L.)

**Keywords:** self-healing, organic/inorganic hybrid materials, biobased copolymer, Diels–Alder chemistry, electromagnetic interference shielding

## Abstract

In this study, a novel biobased poly(ethylene brassylate)-poly(furfuryl glycidyl ether) copolymer (PEBF) copolymer was synthesized and applied as a structure-directing template to incorporate graphene and 1,1′-(methylenedi-4,1-phenylene)bismaleimide (BMI) to fabricate a series of self-healing organic/inorganic hybrid materials. This ternary material system provided different types of diene/dienophile pairs from the furan/maleimide, graphene/furan, and graphene/maleimide combinations to build a crosslinked network via multiple Diels–Alder (DA) reactions and synergistically co-assembled graphene sheets into the polymeric matrix with a uniform dispersibility. The PEBF/graphene/BMI hybrid system possessed an efficient self-repairability for healing structural defects and an electromagnetic interference shielding ability in the Ku-band frequency range. We believe that the development of the biobased self-healing hybrid system provides a promising direction for the creation of a new class of materials with the advantages of environmental friendliness as well as durability, and shows potential for use in advanced electromagnetic applications.

## 1. Introduction

During the past decade, the development of self-healing substances has become an important research field since these smart materials bear resemblance to the living organisms which possess an intrinsic capability to repair their architectural damage via an automatic process without additional intervention. So far, several approaches have been developed to efficiently provide the self-healing property to fabricated materials, such as microcapsules [1,2,3], microvasculars [4,5,6] and nanoreservoirs [7,8]. For these “autonomous” systems, the crack formation ruptures the microcontainers, releasing the liquid healing agents to fill the damage zones and restore the original loading capacity. The healing agents subsequently cure upon reacting with the embedded catalyst, repairing the defects. However, even though the utilization of healing agents encapsulated in hollow storages offers effective function for the healing of cracks, the irreversible healing mechanism of these material systems can heal the cracks at the same position only once because of the consumption of the captured healing agents [9,10,11]. In order to overcome this disadvantage and increase the self-repairing times without external healing agents, reversible self-healing techniques involving the repeat cycles of scission/reformation of chemical bonds have been recently developed [12,13,14,15,16,17,18,19,20,21]. Various stimuli including irradiation, thermal heating, or magnetism could be applied for inducing stimuli-response to repeatedly break and heal the molecular structures of the materials to achieve the self-healing functionality.

Among these reversible self-healing methodologies, a dynamic-covalent bond technology based on Diels–Alder chemistry has received a great deal of attention since the deformation of the crosslinked molecular structure can be easily triggered via a heating process, resulting in a liquification of the polymer material to rebuild the crack area. In most cases, DA type self-healing materials were prepared by combining furan and maleimide functional groups serving respectively as diene and dienophile moieties to form a thermally reversible crosslinked network through a [4 + 2] cycloaddition reaction [22,23,24,25,26,27,28,29,30,31,32]. Healing can be easily achieved by a reverse DA reaction occurring in the temperature range from 100 to 150 °C. In addition to the superior healing efficiency, furan-maleimide DA systems also possess less moisture sensitivity than hydrogen bond systems, showing benefits for various practical applications. Although the progress of DA based materials is increasing rapidly, it can be observed that most researchers primarily focus on molecular design, synthesis routes, and self-healing behavior. In order to further diverse the functionality and broaden the application field of the DA type systems, several important properties could be further introduced. For example, conductivity is greatly required for electronic applications such as microelectronics packaging [33], medical devices [34], artificial skins [35], and electromagnetic interference (EMI) shielding [36]. So far, several research studies have been performed to develop DA based nanocomposites, wherein conductive fillers such as metallic nanoparticles [37], carbon nanotubes [38,39,40], and graphene [41,42,43] were incorporated into the material systems. In particular, graphene has attracted a great interest because this two-dimensional carbon allotrope possesses large aspect ratio as well as superior carrier mobility, and can thereby serve as a promising material in EMI shielding technology [43,44,45]. However, the introduction of graphene sheets into polymers often suffers from serious aggregations that strongly hamper the fabrication of homogeneous composites. Therefore, controlling the dispersity of graphene in polymeric matrix is continuously a hot issue to optimize the internal structure of the DA based hybrids.

Recently, increasing attention is given to sustainable chemistry with the goal to reduce dependence on fossil feedstock and develop alternative polymers from renewable biomass sources [46,47,48]. In this regard, it is anticipated that introducing biobased materials as building blocks to fabricate self-healing hybrid materials would further facilitate the functional systems to take advantage of economic benefit and environmental friendliness, providing a promising direction for creating new class of materials. In this study, we report on the development of a novel self-repairable organic/inorganic hybrid system, wherein a novel poly(ethylene brassylate)-poly(furfuryl glycidyl ether) copolymer (PEBF) was utilized as a structural template to co-assemble graphene and 1,1′-(methylenedi-4,1-phenylene)bismaleimide (BMI). Ethylene brassylate was chosen for the hybrid system not only due to its acquisition from renewable resources but also due to the reactivity to efficiently copolymerize with a furfuryl glycidyl ether monomer which can be used for DA click chemistry. Additionally, graphene being combined with furan in PEBF or maleimide in BMI could act as a dienophile or diene to undergo DA reactions [49]. Therefore, the ternary material system provided interlaced diene/dienophile combinations to efficiently induce a thermally reversible crosslinked network with uniformly distributed graphene sheets via multiple DA reactions. Upon a thermal treatment, the DA crosslinked network could be deformed to liberate the chains via a retro-DA reaction, thus increasing the chain mobility of the PEBF copolymer to give the hybrid system a self-healing ability. Furthermore, the PEBF/graphene/BMI hybrids were endowed with EMI shielding properties, owing to the internally contained conductive graphene sheets. Thus, the organic/inorganic hybrid materials developed in the current study can be used as self-healing protection layers for EMI shielding applications.

## 2. Materials and Methods

### 2.1. Materials

Graphene with a specific surface area ≥15 m^3^/g and size distribution of 5.6 to ~18 μm was supplied by Taiwan Graphene Co., Ltd. (Taipei, Taiwan). Ethylene brassylate (EB, ≥95%) and furfuryl glycidyl ether (FGE, 96%) were purchased from Sigma-Aldrich (St. Louis, MO, USA) and Acros (Geel, Belgium), respectively. These two monomers were used as received. Furfuryl alcohol (FAL, Sigma-Aldrich, 98%), 1,5,7-triazabicyclo[4.4.0]dec-5-ene (TBD, Sigma-Aldrich, 98%) and 1,1′-(methylenedi-4,1-phenylene)bismaleimide (BMI, Sigma-Aldrich, 95%) were purchased and used as received. Additionally, all the solvents for this study were purchased from Sigma-Aldrich and used without any purification.

### 2.2. Methods

#### 2.2.1. Synthesis of Poly(ethylene brassylate)-Poly(furfuryl glycidyl ether) Copolymer (PEBF)

**Figure 1:**
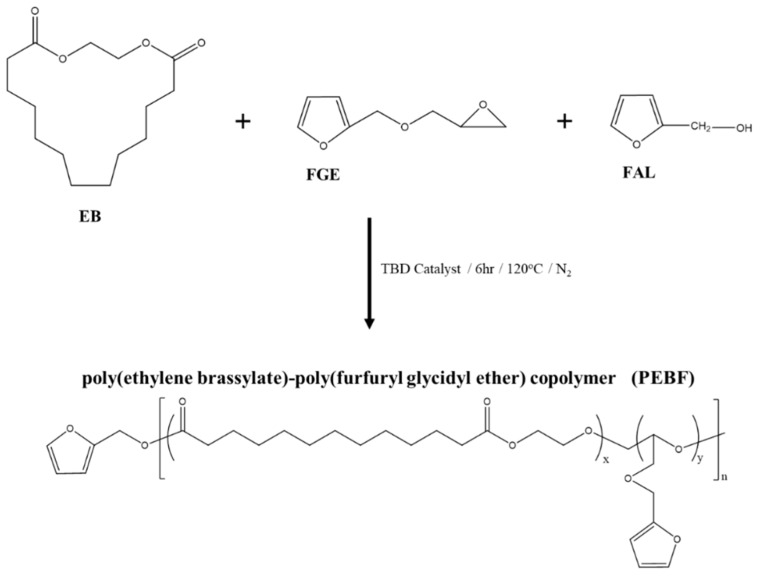
Synthesis of poly(ethylene brassylate)-poly(furfuryl glycidyl ether) copolymer (PEBF) via ring-opening polymerization.

Figure 1 shows the synthesis route of PEBF copolymer. First, EB, FGE, and TBD catalyst were added in a round bottom flask and the mixture was stirred for 2 h under a nitrogen atmosphere at 70 °C. Subsequently, the mixture was heated to 120 °C and then furfuryl alcohol was injected into the flask to initiate the copolymerization reaction. The reaction mixture was stirred at 120 °C to allow the reaction to proceed. After 6 h, the copolymerization was terminated by cooling to room temperature. The crude polymer sample was then dissolved in chloroform, followed by precipitation in ethanol. The desired product was finally obtained by removing the residual solvent by drying in a vacuum oven at room temperature for 24 h.

#### 2.2.2. Preparation of Self-Healing PEBF/Graphene/BMI Hybrid Material

First, a PEBF solution (10 mg mL^−1^) was prepared by dissolving the copolymer in THF solvent, followed by mixing with graphene in different weight fractions of graphene to PEBF of 1 wt %, 2 wt % and 5 wt %. The mixtures were then subjected to a bath sonication for 12 h. Afterward, BMI was added to the PEBF/graphene solutions based on a molar ratio of furan group to maleimide group of 1:1. After mixing for 24 h, the solutions were dried in ambient condition for 48 h. The obtained bulk samples were then placed in a vacuum oven and heated to 180 °C. After heating at 180 °C for 2 h, the hybrid samples were subsequently cooled to 60 °C and then isothermally annealed at 60 °C for 24 h. In addition to the PEBF/graphene/BMI hybrid samples, a self-healing PEBF/BMI sample without graphene was also prepared by following the similar route of the PEBF/graphene/BMI hybrids.

#### 2.2.3. Characterization Methods

For identifying the molecular structure of the synthesized polymer, ^1^H-NMR spectrum of the PEBF copolymer was recorded using a Bruker Fourier 300MHz NMR (Bruker, Billerica, MA, USA) with deuterated chloroform as the solvent. To analyze the molecular weight characteristics of the PEBF copolymer, size exclusion chromatography (SEC) analysis was conducted using a Viscotek TDA 305 system (Malvern, Cambridge, United Kingdom) equipped with a refractive-index detector. THF solvent was used as the mobile phase with a flow rate of 1.0 mL/min, and the measurement was operated at a temperature of 35 °C. Raman analyses were performed by using a Dongwoo Ramboss 500i Micro-Raman/PL spectroscope (DongWoo Optron, Gwangju-si, Korea). For the Raman measurements, thin film samples were prepared by depositing hybrid solutions onto silicon wafer substrates, followed by drying in air for 12 h. The morphologies of the samples were investigated by using a Hitachi H-7650 transmission electron microscopy (Hitachi, Tokyo, Japan) operating at 75 kV. ATR–FTIR spectra were recorded on a Jasco FT/IR-4600 spectrometer (Jasco Corporation, Tokyo, Japan), and flat bulk samples were used for the measurements. Wide-angle X-ray scattering (WAXS) measurements were carried out on beamline 13A1 of the National Synchrotron Radiation Research Center (NSRRC). Bulk samples with 1 mm thickness were used for the WAXS measurements and the incident X-ray was configured with a wavelength of 1.033 Å. TGA measurements were performed on a Hitachi STA7200 instrument (Hitachi, Tokyo, Japan) to analyze the thermal stability of the hybrid system. The sample was heated from 50 to 600 °C at 10 °C/min under nitrogen atmosphere. The thermal properties of the hybrid system were also characterized using a TA Q20 differential scanning calorimeter (TA Instruments, New Castle, DE, USA). The measurements were conducted by heating the sample from 50 to 200 °C at 10 °C/min. A Nikon ECLIPSE LV100N POL microscope (Nikon, Tokyo, Japan) with a Linkam THMS600 hot stage (Linkam, Waterfield, UK) was used to image the self-healing behavior of the hybrid system. Additionally, scanning electron microscopy (SEM) micrographs were captured by using a Hitachi TM4000 Plus instrument (Hitachi, Tokyo, Japan) operating at 20 kV accelerating voltage to monitor the morphologies of the hybrid samples. The EMI shielding performance of the hybrid system was analyzed by the waveguide method using a vector network analyzer (Keysight E5071C, (Keysight, Santa Rosa, CA, USA) in Ku-band frequency range (12.4~18 GHz). A rectangular bulk sample with dimensions of 16 mm × 8 mm × 3.5 mm was placed between the waveguide holders, and the S parameters (*S11, S12, S21, S22*) were measured to calculate the EMI shielding effectiveness.

## 3. Results and Discussion

**Figure 2:**
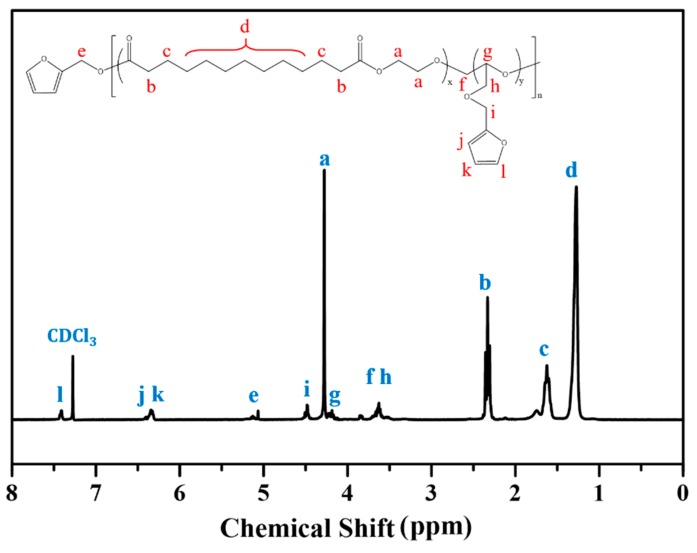
^1^H-NMR spectrum of the synthesized PEBF copolymer in CDCl_3_.

The schematic illustration for synthesizing the PEBF copolymer system is shown in Figure 1. The synthesis was carried out using furfuryl alcohol as an initiator and TBD as a catalyst to copolymerize EB and FGE monomers. To monitor the polymerization of the PEBF copolymer system, the molecular weight characterizations of the synthesized polymer sample were measured by gel permeation chromatography (GPC). From the GPC trace, it could be observed that the obtained polymer had a number-average weight of 6900 g mol^−1^ and a molecular weight distribution of 1.51, indicating the successful synthesis of PEBF. ^1^H-NMR measurement was further performed to characterize the PEBF sample. As shown in Figure 2, the characteristic peaks located at 6.3 and 7.4 ppm corresponded to the protons of furan ring, and the signal at 3.5 to 3.7 ppm was from the protons of opened moiety of FGE. According to the ^1^H-NMR spectrum, the EB to FGE ratio in the PEBF copolymer was estimated to be 77% by calculating the integrated area ratio between the proton signal of furan ring and methylene protons (δ = 2.3 ppm) neighboring the carbonyl group of EB.

**Figure 3:**
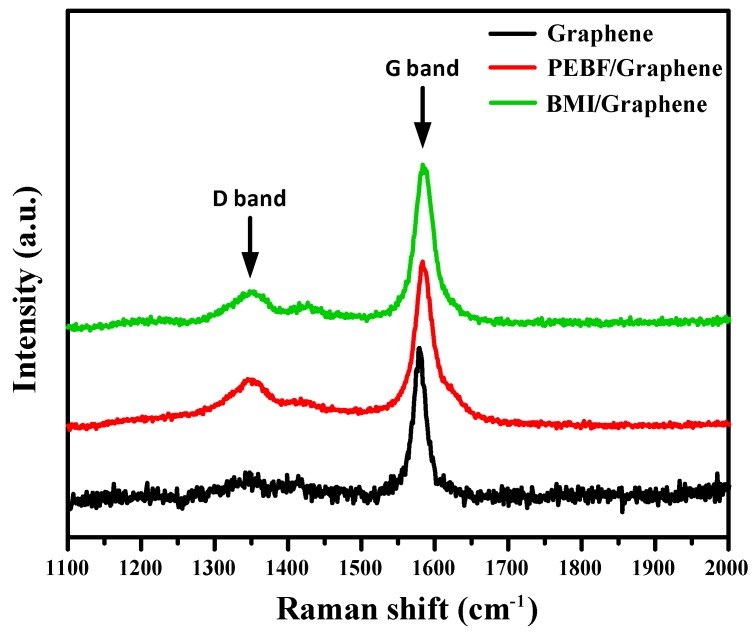
Raman spectra of pristine graphene, PEBF/graphene and 1,1′-(methylenedi-4,1-phenylene)bismaleimide (BMI)/graphene samples.

**Figure 4:**
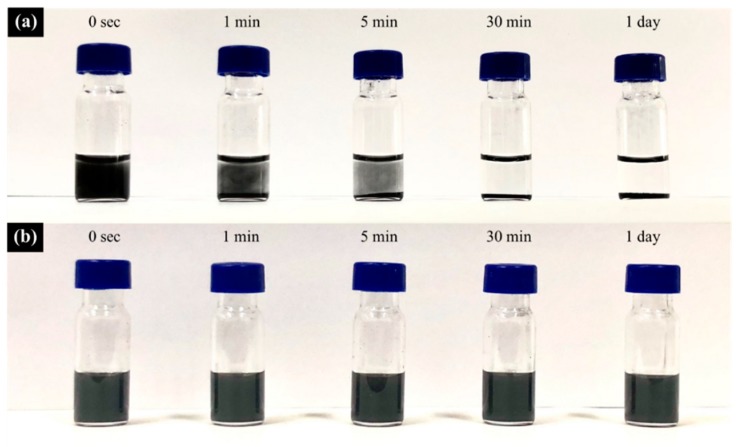
Photographs of (**a**) pristine graphene solutions and (**b**) PEBF/graphene solutions stored for different times.

**Figure 5:**
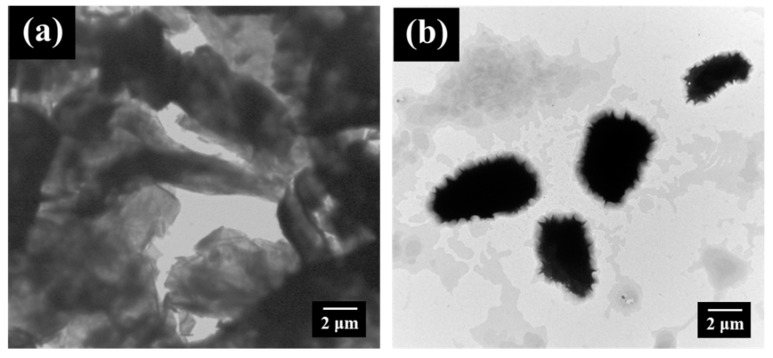
TEM images of (**a**) pristine graphene and (**b**) PEBF/graphene samples.

On the basis of the sample preparation method described above, the self-healing PEBF/graphene/BMI hybrid system was fabricated by first preparing a PEBF/graphene hybrid solution, followed by adding BMI into the solution to produce a ternary material system. Recently, it has been found that carbon materials such as carbon nanotubes, fullerene, and graphene can serve as either a diene material in the [4 + 2] DA cycloaddition with various dienophiles or a dienophile material to react with diene compounds to form DA adducts. Therefore, for the PEBF/graphene hybrid, the furan groups of PEBF could be expected to behave as active diene sites to readily interact with graphene (dienophile) via a DA cycloaddition reaction, resulting in grafting PEBF onto the surface of graphene. For the modified graphene sample, the defect condition and ordered/disordered structures could reveal significant differences from those of the non-modified graphene. Thus, Raman analyses were subsequently performed to study the structural features to confirm the DA reaction between PEBF and graphene. As shown in Figure 3, we observed that the Raman spectra of both the PEBF/graphene hybrid and pristine graphene exhibited a disorder band (D band) at 1350 cm^−1^ and a tangential band (G band) at 1582 cm^−1^. The relative intensity ratio between the D band peak to the G band peak (I_D_/I_G_) corresponded to the bonding transition of graphene from C–C (sp^2^) to C–C (sp^3^), indicating the degree of structural defects on the graphene sample. In contrast to the Raman spectrum of the pristine graphene which exhibited a I_D_/I_G_ value of 0.12, the Raman profile of the PEBF/graphene sample revealed a higher value (I_D_/I_G_ = 0.19). This sudden change indicated the increase of graphene defects resulted from sp^2^ to sp^3^ hybridized carbons, providing the evidence for the occurrence of DA reaction in the PEBF/graphene hybrid. This structural integrity of PEBF attached to the surface of graphene was further confirmed by comparing the solubility of the PEBF/graphene sample and pristine graphene in solvent medium. For the investigations, the PEBF/graphene and pristine graphene were separately added to THF to prepare 0.05 wt % solutions, followed by a sonication for 30 min to disperse these materials in the liquid phase. The prepared solutions were then stored in an ambient environment. The dispersion conditions of these solutions were examined as a function of storage time, as shown in Figure 4. Note that a significant precipitation of graphene could be found in the pristine graphene solution stored for only 1 min (Figure 4a). As the storage time was further increased to 30 min, we could observe that all the graphene sheets precipitated at the bottom of the vial. Comparatively, the PEBF/graphene solution revealed a better dispersibility after 30 min storage (Figure 4b). We could further find that the hybrid solution still exhibited a superior dispersion condition even with a long testing time over 1 day. Figure 5 further shows the TEM images of the pristine graphene and PEBF/graphene samples fabricated by drop coating their THF solutions onto carbon-coated copper grids. We could observe that the morphology of the pristine graphene sample (Figure 5a) showed serious aggregations, indicating that the graphene sheets could not be efficiently dispersed in THF medium. On the contrary, for the PEBF/graphene sample, its TEM image (Figure 5b) revealed a superior dispersion of graphene sheets. It should be noted that the PEBF modified graphene formed an encapsulating structure. The surrounding thin layer with gray contrast could be attributed to the PEBF molecules attached on the surface of graphene. These results of dispersity tests and TEM investigations clearly indicated that graphene sheets were covalently functionalized with PEBF via the DA reaction, consistent with the characteristics of Raman spectra described above.

**Figure 6:**
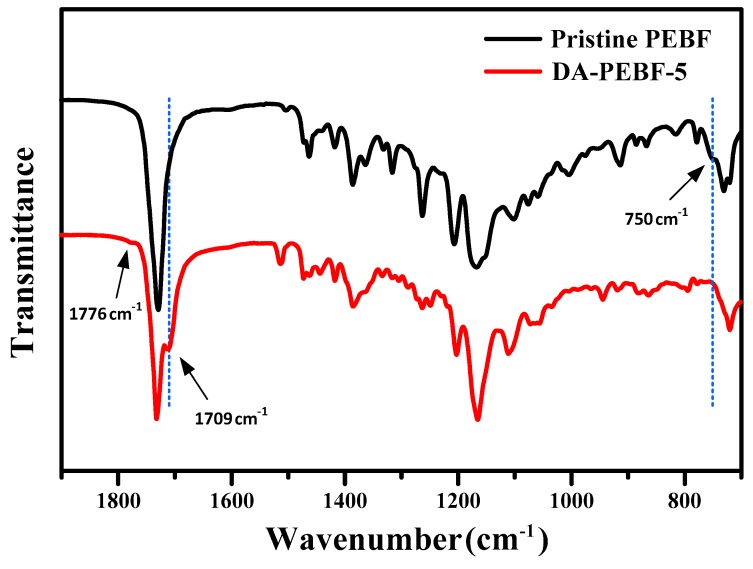
FTIR spectra of pristine PEBF and the DA-PEBF-5 hybrid.

The Raman analysis data and suspension condition of the PEBF/graphene hybrid indicated the successful incorporation of furan functional groups onto graphene surfaces through DA reaction. Furthermore, BMI was added into the PEBF/graphene hybrid to fabricate a PEBF/graphene/BMI ternary system. For the ternary material system, graphene could also serve as a functionalization agent which provided diene sites to react with maleimide groups of BMI (dienophile). Thus formed DA adduct could be also identified by using Raman analysis. For the BMI/graphene hybrid, its Raman spectrum (Figure 3) exhibited a I_D_/I_G_ value of 0.17 higher than that of the pristine graphene sample, showing the evidence for the successful DA reaction. More importantly, the furan groups of PEBF (diene) and maleimide groups of BMI (dienophile) are highly reactive for DA reaction. Therefore, the diene/dienophile pairs from the furan/maleimide, furan/graphene, and maleimide/graphene combinations could efficiently induce multiple DA reactions, resulting in a DA crosslinked network, wherein graphene sheets were uniformly dispersed in the polymeric matrix. In the current study, different amounts of graphene sheets were added to fabricate a series of hybrid samples. The sample designations and corresponding compositional characteristics of the hybrid system are listed in Table 1. The DA mechanism of the PEBF/graphene/BMI hybrid system was characterized by using ATR–FTIR measurements. Shown in Figure 6 are the FTIR spectra of the pristine PEBF and DA-PEBF-5 samples. Compared with the spectrum of the pristine PEBF, the profile of DA-PEBF-5 showed additional peaks located at 1709 and 1776 cm^−1^, which correspond to the C=C stretching vibration of BMI and C=C stretching of DA adduct, respectively. The occurrence of these two signals clearly indicated the formation of DA adducts within the PEBF/graphene/BMI hybrid system. Additionally, the pristine PEBF sample exhibited a shoulder peak at 750 cm^−1^ associated with the signal of furfuryl moiety of the copolymer sample. However, the FTIR spectra of DA-PEBF-5 did not exhibit this shoulder peak, which again proved the formation of DA adducts with the rupture of the original furan rings in PEBF [43].

**Figure 7:**
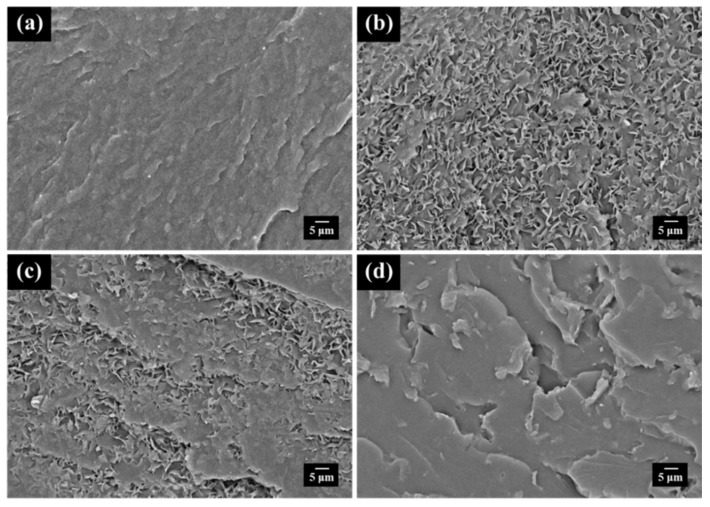
SEM micrographs of fracture surfaces of (**a**) DA-PEBF-0, (**b**) DA-PEBF-1, (**c**) DA-PEBF-2, and (**d**) DA-PEBF-5, respectively.

**Figure 8:**
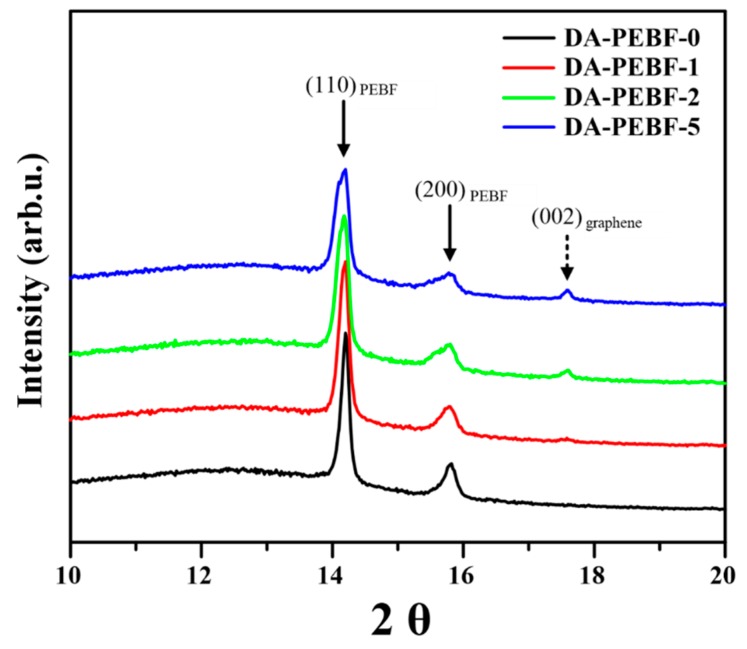
WAXS patterns of DA-PEBF-0, DA-PEBF-1, DA-PEBF-2, and DA-PEBF-5. The solid arrows correspond to the (**110**) and (**200**) crystallographic planes of EB segments in PEBF, and the dashed arrow indicates the (**002**) reflection of graphene.

To further understand the dispersion of graphene sheets in the hybrid system, SEM measurements for monitoring the fracture surfaces of the nanocomposites were subsequently carried out. For the DA-PEBF-0 sample, its SEM micrograph (Figure 7a) showed a typical fracture surface wherein some cracks elongated along the direction of fracturing force. In contrast, the DA-PEBF-1 sample revealed a quite different fracture image, as shown in Figure 7b. An uniform dispersion of graphene sheets without serious agglomerations could be observed, providing strong evidence that the graphene sheets efficiently interacted with the furan groups of PEBF through a DA reaction. Upon increasing the loading amount of graphene to 5 wt % (DA-PEBF-5), the high content of graphene gave rise to a continuous distribution of graphene sheets within the polymeric matrix because of the contacts of these two-dimensional materials. The corresponding fracture surface (Figure 7d) showed a delamination structure with irregular features, indicating an occurrence of crack deflection resulted from the contacted graphene sheets. During the propagation stage, a crack could be twisted or tilted while it encountered the rigid inclusion of graphene, resulting in the formation of the delamination structure. Additionally, the SEM micrograph of DA-PEBF-2 (Figure 7c) showed a coexisting morphology of dispersed graphene sheets and delamination structure since DA-PEBF-2 possessed an intermediate value of graphene content between those of DA-PEBF-1 and DA-PEBF-5. For the hybrid system, the crystallography features were further determined by WAXS measurements. As shown in Figure 8, it could be observe that the WAXS profile of DA-PEBF-0 exhibited two diffraction peaks at 2θ = 14.2° and 15.8°, which correspond to the (110) and (200) crystallographic planes of pristine poly(ethylene brassylate) (PEB), respectively [50]. This result indicated that the DA crosslinked network did not strongly inhibit the crystallization of the EB segments. For the DA-PEBF-1, DA-PEBF-2, and DA-PEBF-5 samples, the (110) and (200) diffraction peaks of PEB were also revealed in the WAXS patterns. It should be noted that there was a reflection signal located at 2θ = 17.6°, which could be assigned to the (002) plane of graphene [51]. Upon increasing the graphene content, the WAXS profiles of these samples showed a progressively decreasing intensity of the PEB crystalline peaks as well as a gradually increased signal of graphene. These results indicated that the added graphene sheets were efficiently co-assembled into the polymeric matrix through the DA reactions, and the covalently bonded fillers could perturb the crystallization behavior of the EB segments in the hybrid system.

**Figure 9:**
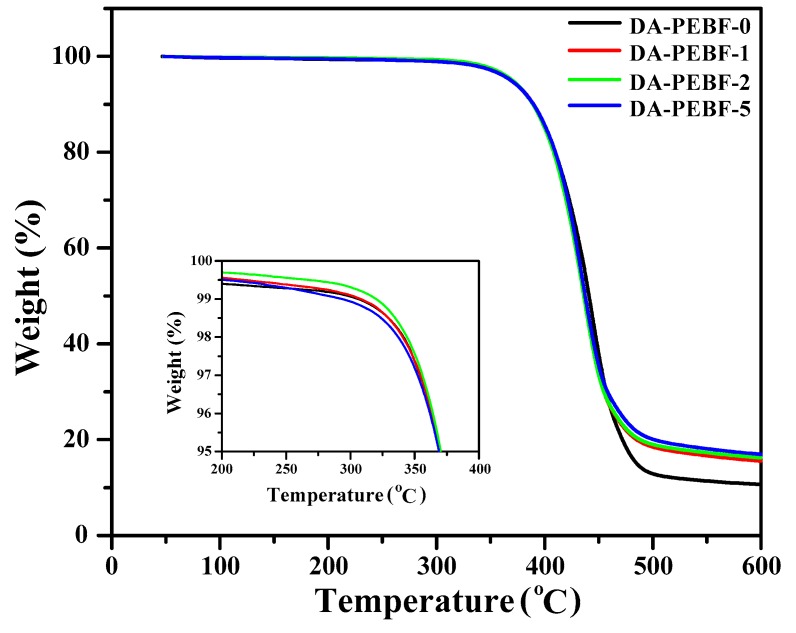
TGA profiles of DA-PEBF-0, DA-PEBF-1, DA-PEBF-2, and DA-PEBF-5 samples. The inset figure shows the detail thermograms within the weight percent range from 95% to 100%.

**Figure 10:**
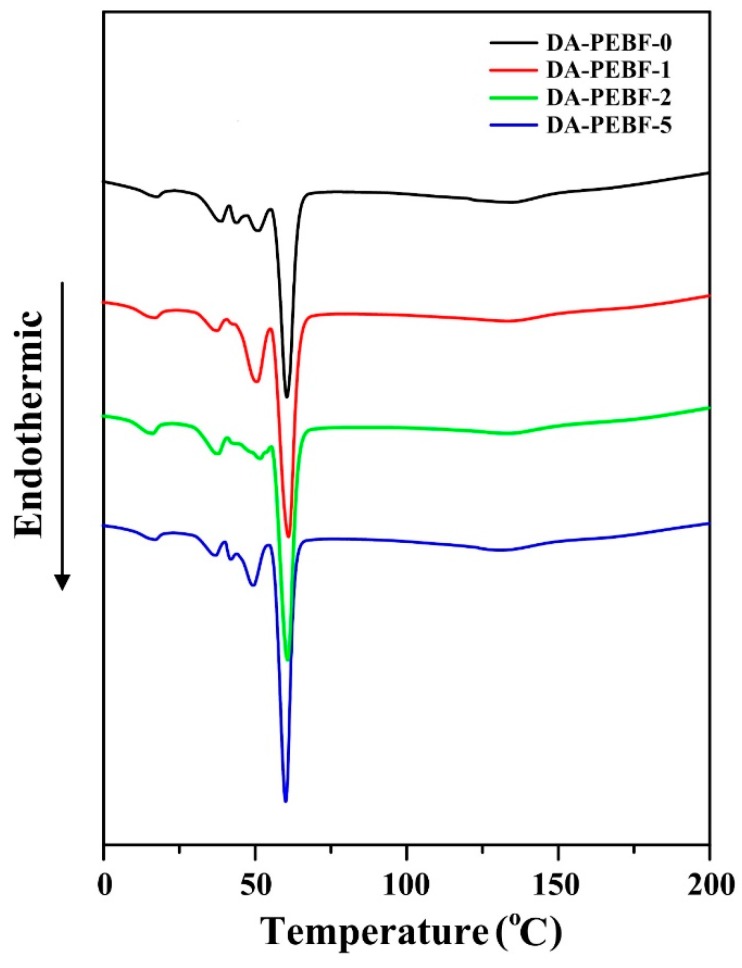
DSC profiles of DA-PEBF-0, DA-PEBF-1, DA-PEBF-2, and DA-PEBF-5 samples.

We subsequently studied the thermal stability of the PEBF/graphene/BMI hybrids. The TGA thermograms of the composite samples with different amounts of graphene sheets can be seen in Figure 9. From the TGA curves, it was found that the pristine PEBF sample and all the composites revealed one stage of decomposition, which could be due to the degradation of the organic parts. The residual mass at 600 °C slightly enhanced with increasing graphene loadings, indicating the successful incorporation of graphene sheets consistent with the results of the WAXS measurements. Additionally, the DA-PEBF-0, DA-PEBF-1, DA-PEBF-2, and DA-PEBF-5 samples all exhibited an initial thermal decomposition temperature (defined as 5% weight loss) higher than 365 °C. The temperatures of 5 wt % mass loss and char residues of these samples are summarized in Table 2. DSC measurements were further used to analyzed the thermal properties of the hybrid system. The DSC thermograms of the PEBF/graphene/BMI samples are shown in Figure 10. For the DA crosslinked hybrids, there were several board spreading endothermic signals appearing in the temperature range between 10 and 70 °C in the DSC thermograms. This observation could be ascribed to the melting signals of non-uniform distribution of crystal sizes induced from the crystallization of the EB segments under the influence of the restricted effect of the DA crosslinked network. For the crosslinked PEBF/graphene/BMI hybrid system, the randomly distributed DA linkages served as fixed anchors on the PEBF chains and strongly disrupted the crystallization of the polymer, thereby resulting in the crystals with a board size distribution. We further observed that the DSC curves of the DA crosslinked samples exhibited a broad endothermic signal in the temperature range between 90 and 180 °C. This signal could be explained by a retro-DA reaction that involved the cleavage of covalent bonds of the DA adducts. In particular, the DSC thermograms of DA-PEBF-1, DA-PEBF-2, and DA-PEBF-5 possessed a combined retro-DA signal from the DA adducts formed from the furan/maleimide, furan/graphene, and maleimide/graphene combinations. The onset temperature and heat flow of the retro-DA reaction of these samples are summarized in Table 2. It could be gathered from the retro-DA thermal features that there was no apparent change in the onset temperature while the heat flow was progressively enhanced with the increment of graphene loading. The gradually increased signal of retro-DA heat flow clearly demonstrated that the introduction of graphene sheets could synergistically increase reactive diene sites as well as dienophile groups to form additional DA linkages, and thus efficiently improved the DA crosslinking density of the hybrid system. For debonding, the reinforced DA crosslinked structure, increased endothermic heat was needed.

**Figure 11:**
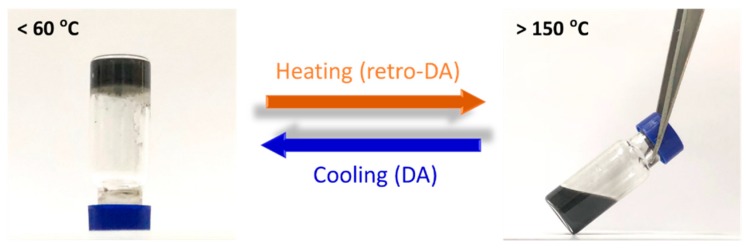
Photographic images taken from a DA-PEBF-2/DMSO mixture (60 wt % in DMSO) under a heating/cooling cycle, demonstrating a thermally reversible sol–gel transformation through DA/retro-DA reactions.

**Figure 12:**
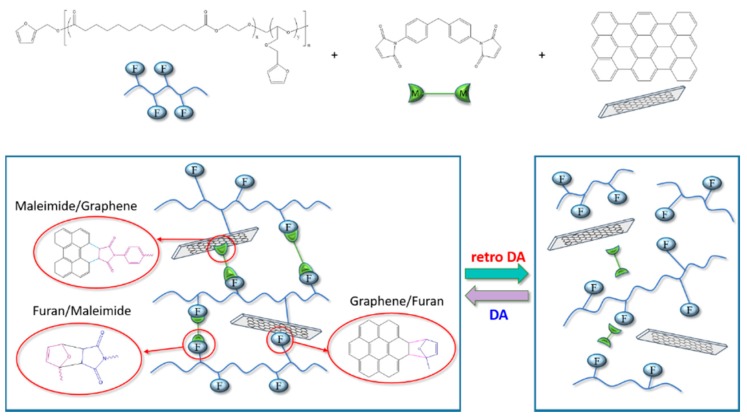
Schematic illustration for the crosslinked structure of the PEBF/graphene/BMI hybrid. This ternary material system offered different diene/dienophile pairs from the furan/maleimide, graphene/furan, and graphene/maleimide combinations to readily induce multiple DA reactions to form a crosslinked network with an uniform distribution of conductive graphene fillers.

To further verify the thermal reversibility of the PEBF/graphene/BMI hybrid system, a sol–gel experiment was used. This investigation was taken with a solid/liquid mixture prepared by mixing DA-PEBF-2 with DMSO solvent (60 wt % in DMSO). Shown in Figure 11 were the images recorded from the recycling study. Upon heating the mixture above 150 °C, it could be observed that the crosslinked DA-PEBF-2 sample dissolved in DMSO medium, resulting in a hybrid solution with good liquidity. This dissolution phenomena clearly demonstrated the disconnection of the DA linkages of DA-PEBF-2 via the thermally triggered retro-DA reaction. The liberation of the molecular chains and graphene sheets induced the phase transition of the hybrid sample from the original crosslinked solid to a plastic state, thereby increasing the miscibility with DMSO. Upon cooling the solution to a temperature lower than 60 °C, a significant solution to gel transition was observed, indicating an efficient recovery of the DA crosslinked network. Furthermore, the sol–gel transformation could appear repeatedly upon multiple heating/cooling cycles, reflecting the ability of the hybrid system to undergo thermally reversible DA cycloaddition reactions. Based on the combined results of Raman, TEM, FTIR, SEM, WAXS, DSC, and sol–gel experiments, a schematic illustration which shows the structure evolution and thermal reversibility of the PEBF/graphene/BMI hybrid system is shown in Figure 12.

**Figure 13:**
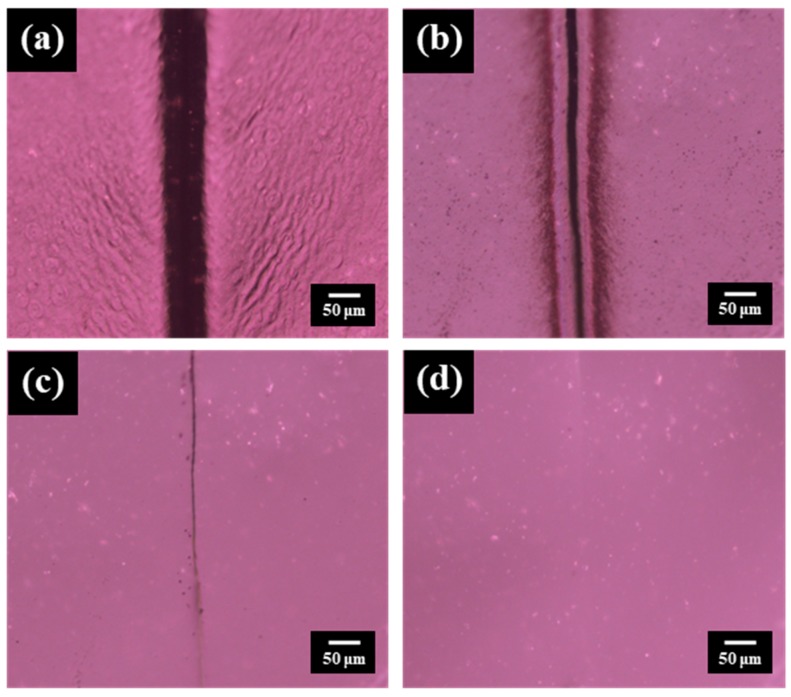
Optical microscope images recorded from the self-healing tests on a scratched DA-PEBF-2 sample at (**a**) room temperature, (**b**) 60 °C, (**c**) 120 °C and (**d**) 140 °C, respectively.

**Figure 14:**
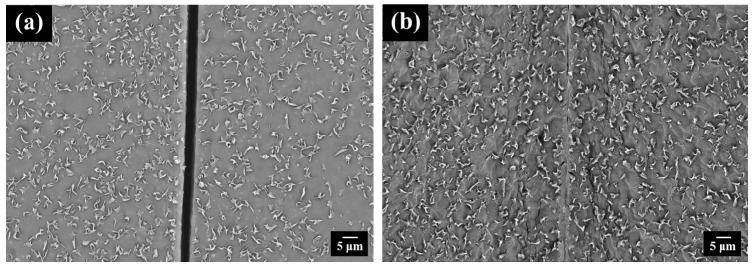
SEM images of a micro-scratched DA-PEBF-2 sample (**a**) before and (**b**) after self-repair.

Due to the thermally reversible feature of DA mechanism, the PEBF/graphene/BMI hybrid system was expected to have self-healing ability for repairing structural damages. For evaluating the self-healing performance of this system, a rectangular bulk sample with a cut of 1 mm depth on its surface was first prepared, followed by monitoring the morphological change of the cracked sample under different heating temperatures by using an optical microscopy. These measurements were started from room temperature with a heating step of 10 °C. At each temperature, the measured sample was isothermally heated for 10 min to cause the surface morphology of the sample to reach an equilibrium state. Shown in Figure 13a–d are the representative OM micrographs recorded from the measurements. For the scratched sample at room temperature, its OM image showed a visible knife mark on the surface. As the sample was heated to 60 °C, the scratch slightly disappeared but could still be observed. The slight healing effect could be attributed to the melting of the crystalline EB segments. With increasing the temperature, we could observe that the partially healed scratch showed no visible change in the temperature region from 80 to 110 °C, reflecting that the DA crosslinked network still supported the internal structure of the material and significantly restricted further motion of the molecules. When the sample was heated from 110 to 120 °C, the remaining scratch started to disappeared, indicating that the crosslinked network of the hybrid dissociated into individual building components through a retro-DA reaction, resulting in an increased chain mobility of the polymer matrix that allowed sufficient molecular diffusion to cure the crack site. Upon further heating to 140 °C, a complete closure of the cut could be obtained, demonstrating the self-repairing ability of the hybrid system. The detail information demonstrating the crack image versus healing time at different temperatures is shown in Figure S1. The crack repair of the hybrid system in microscale was further investigated by SEM measurements. Shown in Figure 14a,b were the SEM images of a micro-scratched sample before and after healing. These micrographs also revealed efficient self-healing behavior, relating well with the results from OM measurements.

The EMI shielding performance of the hybrid system was further quantified as the relative intensity ratio between the incident electromagnetic wave to the transmitted wave. The total attenuation of the incident electromagnetic wave can be expressed as the following equation:

SE_T_ (dB) = SE_A_ + SE_R_ + SE_M_ (1)

where SE_T_ is the total EMI shielding effectiveness, SE_A_ is the absorption of electromagnetic wave, SE_R_ is the reflection from the material surface and SE_M_ is the multiple reflections occurred at the inhomogeneous interfaces within the material [52,53]. Note that the item of multiple reflections can be included with the part of absorption since the multi-reflected waves within EMI shielding material can be absorbed and then dissipated as heat, similar to the mechanism of absorption [54]. Thus, the total shielding effectiveness can be simplified as follows:

SE_T_ (dB) = SE_A_ + SE_R_ (2)

**Figure 15:**
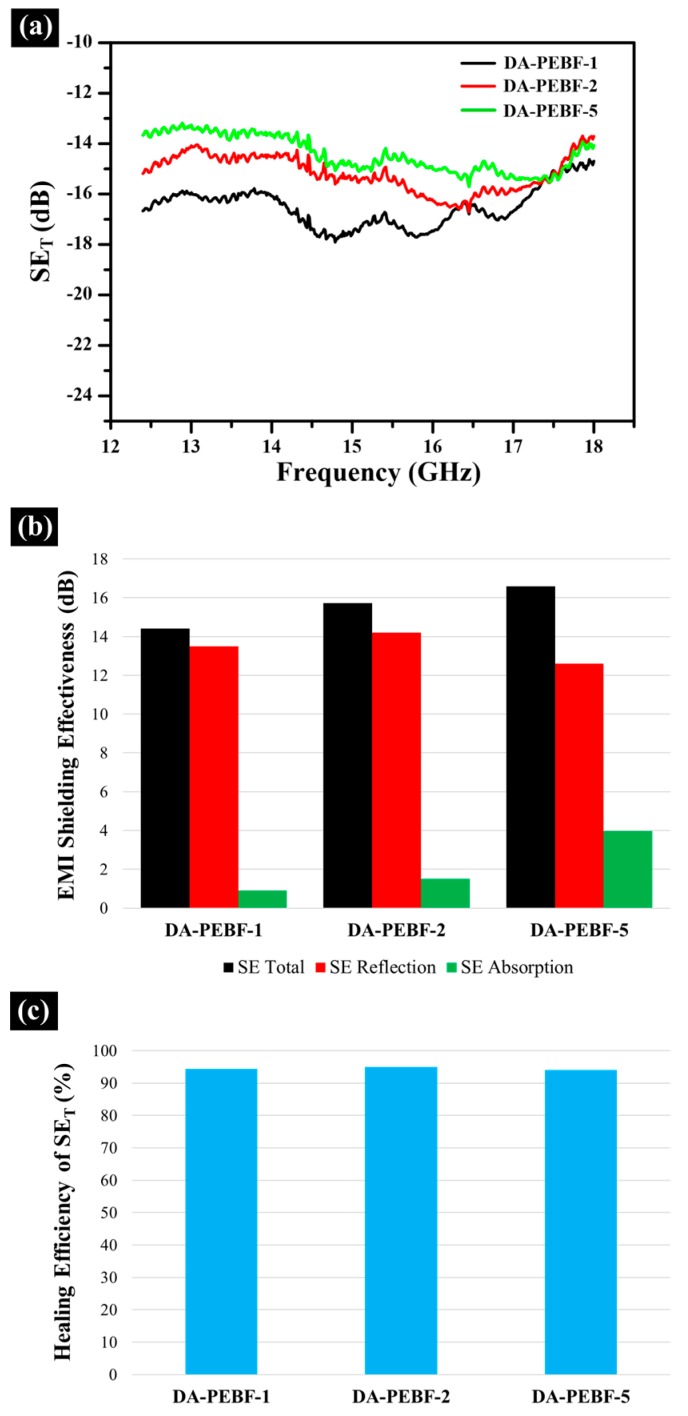
(**a**) SE_T_ profiles of PEBF/graphene/BMI hybrid samples with different graphene loadings as a function of frequency in Ku-band. The average values of SE_T,_ SE_A_, and SE_R_ are further summarized in a bar graph, as shown in (**b**). The healing efficiency of SE_T_ of the hybrid samples are shown in (**c**).

The variation in total EMI SE in Ku-band (12.4~18 GHz) as a function of different amounts of graphene for the PEBF/graphene/BMI hybrid samples is presented in Figure 15a. In order to clarify the detailed mechanism of the EMI shielding performance, the average values of SE_T,_ SE_A_, and SE_R_ in Ku-band are further summarized in Figure 15b. The DA-PEBF-1 sample had an average SE_T_ of 14.4 dB. Additionally, the SE_A_ and SE_R_ of DA-PEBF-1 are measured to be 0.9 and 13.5 dB. From these results, we could observe that the reflection efficiency mainly provided a major contribution to the attenuation of the incident electromagnetic microwave rather than the absorption efficiency. The reflection of electromagnetic waves could be attributed to the impedance mismatch between air and the hybrid system with uniformly dispersed conductive graphene sheets. As for incorporating 2 wt % graphene into the hybrid (DA-PEBF-2), we could observe that the DA-PEBF-2 sample exhibited a SE_A_ of 1.5 dB, a SE_R_ of 14.2 dB, and gave a SE_T_ of 15.7 dB higher than that of the DA-PEBF-1 sample. Furthermore, an improvement in the EMI shielding performance was achieved in the DA-PEBF-5 sample, showing a SE_A_ of 4.0 dB, a SE_R_ of 12.6 dB, and a SE_T_ of 16.6 dB. As a comparison, it could be clearly observed that the SE_A_ value gradually rose with the increasing graphene fraction while the level of SE_R_ only showed a tiny variation. The changing trend of EMI shielding performance of the PEBF/graphene/BMI system could be attributed mainly to the different microstructures of the hybrid samples. Based on the SEM results discussed above, there was a structural change from a uniform dispersion of graphene sheets to a continuous graphene distribution as the graphene content was increased from 1 to 5 wt %. The gradually contacted graphene sheets could form interconnected networks in the polymeric matrix and provided the ability to restrict the incident electromagnetic microwaves from escaping from the compartments and caused the waves to be multireflected and absorbed [55], leading to an improvement of the electromagnetic wave absorption.

The EMI shielding performances of the original and healed hybrid samples were further evaluated. Before the self-repairing process, rectangular hybrid samples (16 mm × 8 mm × 3.5 mm) were cut to form a large crack with a width of 1 mm and depth of 2 mm. From the experimental results, it could be observed that the self-repairability for healing the structural defect simultaneously resulted in a promising restoration of SE_T_ to at least 94% of the initial value (shown in Figure 15c), demonstrating that the PEBF/graphene/BMI hybrid system exhibited adequate self-healing capacity for healing structural defects as well as recovering intrinsic properties.

## 4. Conclusions

In summary, we have successfully synthesized a novel biobased PEBF copolymer and fabricated a PEBF/graphene/BMI hybrid material system by incorporating graphene sheets and BMI into the polymeric matrix. Beside the reactive furan-maleimide combination, graphene could also serve as an effective DA crosslinking agent to build the DA crosslinked network. For this hybrid system, the DA adducts induced from multiple diene/dienophile combinations and their thermal reversibility were systematically analyzed by Raman, TEM, FTIR, SEM, WAXS, TGA, DSC, and sol–gel measurements. This organic/inorganic hybrid system provided multiple functions including self-repairability as well as EM wave shielding ability. Additionally, the use of biobased monomer ethylene brassylate further showed the benefits of economics and environmental friendliness. Therefore, this research provides a new way for developing advanced biobased materials with self-repairing and EMI shielding functionalities, which could be applied as self-healing protection layers for EMI shielding applications.

## Figures and Tables

**Table 1:** Compositional characteristics of PEBF/graphene/BMI hybrids.

Sample Code ^1^	FGE/BMI Ratio ^2^	Graphene Amount ^3^
DA-PEBF-0	1:1	0 wt %
DA-PEBF-1	1:1	1 wt %
DA-PEBF-2	1:1	2 wt %
DA-PEBF-5	1:1	5 wt %

^1^ Designations of the hybrid samples. ^2^ The molar ratio of FGE monomer units to BMI. ^3^ The added weight ratio of graphene to PEBF copolymer.

**Table 2:** Thermal properties of PEBF/graphene/BMI hybrids with different loading of graphene.

Sample ^1^	5wt % Loss Temperature (°C) ^2^	Char Residue at 600 °C (%) ^3^	Retro-DA Onset Temperature (°C) ^4^	Retro-DA Heat Flow (J/g) ^5^
DA-PEBF-0	369	10.6	95	8.0
DA-PEBF-1	369	15.5	94	9.8
DA-PEBF-2	370	16.2	95	11.4
DA-PEBF-5	368	16.9	96	16.5

^1^ Designations of the hybrid samples. ^2,3^ 5 wt % loss temperature and char residue at 600 °C were identified from TGA profiles. ^4,5^ Onset temperature and endothermic heat flow of retro-DA reaction were determined from DSC thermograms.

## Supplementary Materials

The following are available online at https://www.mdpi.com/2073-4360/11/11/1755/s1, Figure S1: Detail information demonstrating crack image versus healing time at different temperatures.
