# Supplementary material for: Development of Self-Healable Organic/Inorganic Hybrid Materials Containing a Biobased Copolymer via Diels–Alder Chemistry and Their Application in Electromagnetic Interference Shielding
Source: Polymers (Basel). 2019 Oct 25;11(11):1755. doi: 10.3390/polym11111755 (PMC6918365; doi:10.3390/polym11111755)
Supplement: Supplementary file 1 [file polymers-11-01755-s001.pdf]

Supporting Information

Development of Self-Healable Organic/Inorganic Hybrid Materials Containing Biobased Copolymer via Diels-Alder Chemistry and Their Application in Electromagnetic Interference Shielding

Yi-Huan Lee [1,2,3,*], Wen-Chi Ko [1], Yan-Nian Zhuang [1,3], Lu-Ying Wang [1], Tao-Wei Yu [4], Shaio-Yen Lee [4], Tun-Fun Way [3] and Syang-Peng Rwei [1,2,3]

[1] Institute of Organic and Polymeric Materials, National Taipei University of Technology, Taipei 10608, Taiwan; asd14120asd@gmail.com (W.-C.K.); nickhamesome@gmail.com (Y.-N.Z.); ying3650@gmail.com (L.-Y.W.); f10714@ntut.edu.tw(S.-P.R.)
[2] Department of Molecular Science and Engineering, National Taipei University of Technology, Taipei 10608, Taiwan
[3] Research and Development Center for Smart Textile Technology, National Taipei University of Technology, Taipei 10608, Taiwan; tfway1951@gmail.com (T.-F.W.)
[4] Taiwan Graphene Co. Ltd., Taipei 11493, Taiwan; bob.yu@angstronmaterials.com (T.-W.Y); shaoyen.lee@angstronmaterials.com (S.-Y.L)

*Correspondence: yihuanlee@mail.ntut.edu.tw (Y.-H.L.); Tel.: 886-2-27712171 (ext 2410) (Y.-H.L.)

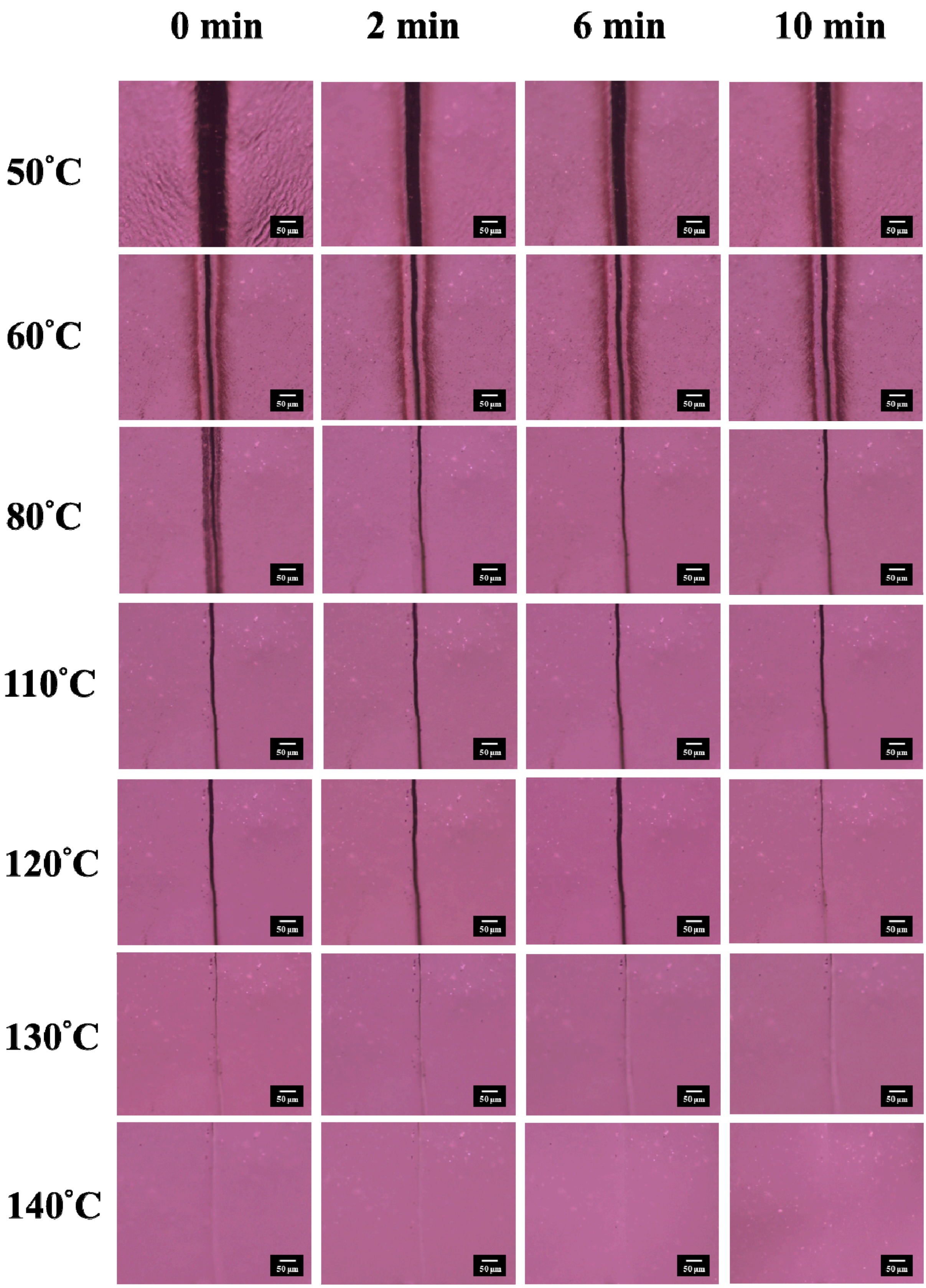

Figure S1. Detail information of optical microscope images recorded from the self-healing tests on a scratched DA-PEBF-2 sample for demonstrating the crack image versus healing time at different temperatures.
